# Sensor for Rapid In-Field Classification of Cannabis Samples Based on Near-Infrared Spectroscopy

**DOI:** 10.3390/s24103188

**Published:** 2024-05-17

**Authors:** Robert Zimmerleiter, Wolfgang Greibl, Gerold Meininger, Kristina Duswald, Günther Hannesschläger, Paul Gattinger, Matthias Rohm, Christian Fuczik, Robert Holzer, Markus Brandstetter

**Affiliations:** 1 Research Center for Non-Destructive Testing GmbH, Altenberger Straße 69, 4040 Linz, Austria; kristina.duswald@recendt.at (K.D.); guenther.hannesschlaeger@recendt.at (G.H.); paul.gattinger@recendt.at (P.G.); robert.holzer@recendt.at (R.H.); 2 Criminal Intelligence Service, Forensic Science, Josef Holaubek Platz, 1090 Wien, Austria; wolfgang.greibl@bmi.gv.at; 3 Spath Micro Electronic Design GmbH, Reininghausstraße 13, 8020 Graz, Austria; g.meininger@meds.at; 4 IFHA/Christian Fuczik-Chemisches Labor GmbH, Gerhardusgasse 25/3.OG, 1200 Wien, Austria; technik@hanfanalytik.at (M.R.); info@hanfanalytik.at (C.F.)

**Keywords:** cannabis analysis, near-infrared spectroscopy, handheld sensor, law enforcement, partial least squares discriminant analysis, forensic science, machine learning, chemometrics

## Abstract

A rugged handheld sensor for rapid in-field classification of cannabis samples based on their THC content using ultra-compact near-infrared spectrometer technology is presented. The device is designed for use by the Austrian authorities to discriminate between legal and illegal cannabis samples directly at the place of intervention. Hence, the sensor allows direct measurement through commonly encountered transparent plastic packaging made from polypropylene or polyethylene without any sample preparation. The measurement time is below 20 s. Measured spectral data are evaluated using partial least squares discriminant analysis directly on the device’s hardware, eliminating the need for internet connectivity for cloud computing. The classification result is visually indicated directly on the sensor via a colored LED. Validation of the sensor is performed on an independent data set acquired by non-expert users after a short introduction. Despite the challenging setting, the achieved classification accuracy is higher than 80%. Therefore, the handheld sensor has the potential to reduce the number of unnecessarily confiscated legal cannabis samples, which would lead to significant monetary savings for the authorities.

## 1. Introduction

The cannabis plant is one of the oldest cultivated plants and has long been used for production of hemp fiber, hemp seeds and oils as well as medicinal purposes, but also as a recreational drug [1,2]. Consumption of parts of the female cannabis plant, mostly cannabis sativa, cannabis indica, or crossbreeds thereof, can result in an intoxicating effect, mainly due to the plant’s principal psychoactive constituent Δ9-tetrahydrocannabinol (THC). Besides THC, over 100 other, non-psychoactive cannabinoids can be found in the cannabis plant [3]. Cannabidiol (CBD) is one example that has gained increasing popularity in recent years due to its relaxing and pain-relieving effect.

In Austria, cannabis products can legally be produced and sold if the THC content does not surpass a threshold of 0.3 wt%, which is determined by the Austrian authorities using gas chromatography in combination with a flame ionization detector (GC-FID). Due to the increasing popularity of legal cannabis products rich in CBD, Austrian authorities frequently encounter these products on the street, which can’t be distinguished from THC-rich cannabis products via appearance or odor. This results in a high number of confiscated legal cannabis samples, which in turn leads to increased personnel and financial expenditures. This could be avoided via a reliable in-field measurement to distinguish legal from illegal cannabis samples.

Besides laboratory analysis using chromatographic measurement techniques [4,5], alternative methods to determine THC in cannabis are commercially available, e.g., THC test kits [6,7,8] or microelectronic gas sensors [9,10,11]. However, these methods do not provide the necessary measurement precision to reliably distinguish between legal and illegal samples. Nevertheless, optical measurement techniques, which allow measurement of the THC concentration in cannabis, including e.g., near-infrared (NIR) [12,13,14], mid-infrared (MIR) [15], and Raman spectroscopy [16,17], have been presented in the literature. Mostly, published works on THC measurements and commercially available optical cannabis sensors [18,19,20] focus on potency measurements, i.e., measurement of total THC concentration in the investigated samples. Measurement precisions down to a root mean squared error of prediction (RMSEP) of approximately 1.3 wt% [12], and 2.32 wt% [21] THC, have been reported for NIR and MIR spectroscopy, respectively.

While the presented precisions are sufficient to allow for the estimation of potency of different cannabis products, they do not allow for a reliable classification between legal and illegal cannabis products, due to the low threshold of 0.3 wt%. However, it has already been shown that by using classification via partial least squares discriminant analysis (PLS-DA), it is possible to distinguish between legal and illegal cannabis samples, based on their absorption in the NIR spectral range with an accuracy of >90% in a laboratory environment on crushed samples when an average spectrum of five measurement spots per sample is used [22].

Herein, we present a dedicated NIR-based sensor solution for classification of cannabis samples in the field which allows measurement without any sample preparation through commonly encountered, transparent plastic packaging made from polyethylene (PE) or polypropylene (PP), able to reliably classify approximately 90% of all investigated samples with a classification accuracy of over 80% using partial least discriminant analysis (PLS-DA) carried out directly on the sensor hardware.

## 2. Materials and Methods

### 2.1. Cannabis Samples

The cannabis samples investigated in this study were partly provided by the Austrian authorities and partly by the IFHA (Institut für Hanfanalytik) in Vienna. The investigated cannabis sample set was made up from 305 different samples, enveloping a wide range of different types with THC concentrations from 0.02 wt% to 24.38 wt% and CBD concentrations from 0.02 wt% to 18.69 wt% as determined by chromatographic reference measurements. Cannabis samples were partly available as fully intact flower buds and partly as ground powder, with different degrees of fineness of the ground sample as well as different composition (i.e., different ratios of plant materials such as flower buds, stems, and leaves) and were intentionally used as is for the NIR spectroscopic measurement to cover as much variation in the sample composition as possible.

### 2.2. Chromatographic Reference Measurements

Reference values of the investigated samples were provided by the Austrian Criminal Intelligence Service and the IFHA, determined using GC-FID and high-pressure liquid chromatography (HPLC), respectively.

GC-FID measurements were carried out on a GC-2100plus (Shimadzu, Kyoto, Japan) with an AOC 20s autosampler and an AOC 20i split/splitless injector (kept at 290 °C, split ratio 120, 1 µL sample volume) combined with a flame ionization detector (kept at 300 °C). The autosampler tray was cooled to 16 °C with a Minichiller 300 (Peter Huber Kältemaschinenbau SE, Offenburg, Germany). Separation was performed on a quartz capillary HP-5 25 m × 0.32 mm with a film thickness of 0.17 µm (Agilent, Santa Clara, CA, USA) with helium as a carrier gas with a constant flow rate of 1.5 mL/min. The starting temperature of 230 °C was kept for 10 min and subsequently raised at 20 °C/min up to a temperature of 250 °C and further increased to 300 °C, at a rate of 50 °C/min. The end temperature was then kept for 7 min.

The GC-FID method was calibrated with cannabidiol (CBD) powder (Lipomed AG, Arlesheim, Switzerland) because the THC-standard solution (1 mg/mL in ethanol, Lipomed AG, Arlesheim, Switzerland) was very unstable, and it has been shown that CBD can be used as a model substance to improve the accuracy in the quantification of THC [23]. The calibration was checked using the purchased standard THC solutions as well as an in-house cannabis standard and round-robin tests.

Before the GC-FID measurements, cannabis samples coming from police forces were extracted via solid-liquid extraction with a solution of dextromethorphan, used as internal standard, in butyl acetate. Around 200 mg of dried sample was weighed and extracted using 5 mL of the butyl acetate solution, vigorously shaken, and left for 30 min at 40 °C. Samples were then centrifuged for 2 min at 4500 rpm before the clear solution above the plant material was collected and used for the GC-FID analysis. Calibration is re-checked with the in-house standard processed in the same batch run on the GC-FID.

HPLC measurements were carried out on an LC-2040C 3D Plus (Shimadzu, Japan) coupled with an online membrane degasser and autosampler. Detection was carried out using a diode array detector (DAD) and deuterium lamp in the wavelength range 190 nm–800 nm. As solvents, a solution of 5 mmol ammonium formate in water (solvent A) and acetonitrile (solvent B) were used as organic and aqueous eluent, both containing 0.1% formic acid. The ratio of solvent A:B was chosen as 35:65 as starting condition and a flow rate of 1.5 mL/min at a temperature of 30 °C was used for the measurements. The utilized HPLC-DAD method was developed in-house at the IFHA and is based on an isocratic method for quantification of 17 phytocannabinoids in under 11 min [24].

Before conducting the HPLC-measurements, 100 mg of dried cannabis was weighed, and gradient-grade methanol (Honeywell Riedel-de-Haën, USA) was added using a pipetting robot. Subsequently, the samples were vigorously shaken for 15 min before being centrifuged for 5 min at 10,000 rpm. The supernatant above the plant material was then mixed with a ratio of 1:66.7 with gradient-grade methanol, already containing the internal standard (Norgestrel, Analytical Standard > 99 %, Sigma Aldrich, St. Louis, MI, USA), in a 2 mL reaction vessel. 600 µL were then pipetted onto a 0.2 µm Claristep filter (Sartorius, Göttingen, Germany) and filtered into a 1.5 mL HPLC vial.

The estimated measurement uncertainty for both chromatographic reference methods is estimated at 10% of the measured value. Data evaluation for both GC-FID and HPLC was carried out using the software LabSolutions^TM^ 5.71 from Shimadzu.

### 2.3. NIR Spectroscopy

#### 2.3.1. Spectrometer Modules

Spectral data were acquired using low-cost NIR spectrometer modules based on tunable Fabry-Pérot filters (NIRONE-X, Spectral Engines, Steinbach, Germany) in diffuse reflection geometry. The diameter of the measurement spot is approximately 1.3 mm as stated by the manufacturer. Spectra of the different cannabis samples were collected in either direct contact with ground and unground cannabis samples, or through commonly available transparent polymer packages made from PE or PP. A commercially available white reference (Spectral Engines, Germany) was used as background to calculate absorbance spectra of the investigated samples.

Spectral data points were acquired in 5 nm steps in the wavelength range from 1550 nm to 1950 nm, where the main absorption features of THC in the NIR spectral region are encountered, which resulted in a total of 81 data points per NIR spectrum. At each 5 nm step, the measured intensity was averaged 50 times and a total of 20 full scans over the wavelength range were carried out, which results in a total measurement time of approximately 5.5 s per spectrum. Three of these spectra were measured consecutively before being averaged, which provides a means of determining if the sample has moved during the measurement via calculation of the standard deviation of the individual spectra and comparing it to a predefined threshold, while also improving the signal-to-noise ratio (SNR) and still fulfilling the requirement for measurement speed.

A total of five different individual NIRONE-X spectrometer modules were used to acquire the data presented herein, one of which was always utilized as a standalone module with the measurement software provided by the manufacturer, while the other four were incorporated into the developed sensor prototypes (see description in Section 3.1) and controlled via the custom-designed electronics and software.

#### 2.3.2. NIR Data Set

The NIR data set used for calibration of the chemometric models consists of 4260 spectra measured on 305 different samples. Measurements were carried out with 5 different NIR spectrometer modules, to also include module-to-module variations within the data set. The calibration data were partly measured by NIR spectroscopists and partly by employees of the IFHA and the Austrian authorities after a short briefing on how to use the developed sensor prototype.

The independent validation data set contains NIR spectra of 83 different samples where each sample was measured four times—two times with each sensor prototype and, in each case, once in a transparent PE and once in a transparent PP bag. Since every sensor prototype contains two NIR sensor modules, two spectra are measured on each of the samples at different positions. 

For classification, it was decided to use a slightly higher THC threshold of 0.4 wt%, since (i) it makes the available data set more evenly divided between the two classes, and (ii) samples between 0.3 and 0.4 wt% usually do not lead to further investigations and are therefore not a priority to be detected by the handheld cannabis sensor.

### 2.4. Chemometric Data Evaluation

#### 2.4.1. Preprocessing of NIR Spectral Data

Acquired spectra were preprocessed to reduce interference originating from differences in sample composition, such as sample particle size and differences in the overall measured light intensity. After testing several pretreatment methods, it was found that smoothing and first derivative using a Savitzky-Golay filter with a window size of 5 data points and second order polynomial, followed by a Standard Normal Variate (SNV) filtering, was best suited to minimize unwanted interference in the absorbance spectra while preserving relevant chemical information in the NIR spectra of the investigated cannabis samples.

#### 2.4.2. Partial Least Squares Methods (PLS-R, PLS-DA, iPLS)

Partial Least Squares regression (PLS-R) is a supervised chemometric technique widely used in spectroscopic applications to extract continuous responses from acquired spectra, such as concentrations of different analytes in different matrices [25,26].

According to the literature, PLS-R models for THC quantification are typically not precise enough to distinguish between legal and illegal cannabis [12,13]. To confirm this, we trained a PLS-R model on the NIR spectra of the calibration data set and used it to predict the THC levels of the validation set, leading to an RMSEP of 2.0 wt% with an R² of 0.857, comparable to previously reported values. While this prediction quality is sufficient for estimation of the THC potency of the samples, it is obviously not sufficient for reliable discrimination between legal (≤0.3 wt% THC) and illegal (>0.3 wt% THC) cannabis. A suitable alternative for that task is Partial Least Squares Discriminant Analysis (PLS-DA), which is a form of PLS combined with discriminant analysis, that can be used for classification of different samples [27].

Prior to calibration of the PLS-DA chemometric model, the most relevant spectral regions for modelling were determined using interval PLS (iPLS) [28,29], with an interval size of 5 variables (which amounts to 25 nm) on the whole calibration data set using a 90/10 cross-validation. The iPLS algorithm selects a subset of the available variables which results in superior prediction compared to using all the variables in a data set.

All PLS-based calculations (PLS-R, PLS-DA, iPLS) presented herein were carried out using MATLAB R2016b (MathWorks Inc., Natick, MA, USA) equipped with the PLS-Toolbox 8.0.2 (Eigenvector Research Inc., Manson, WA, USA).

## 3. Results

### 3.1. Design of a Portable NIR-Based Sensor for Classification of Cannabis Samples

Together with the Austrian authorities, the most important requirements for a field-applicable sensor solution suitable for quick on-site classification of cannabis samples were defined, and are listed below:

- Little to no sample preparation before the measurement.
- Non-destructive measurement of the sample.
- Measurement must be possible through a transparent plastic bag.
- Measurement time < 20 s.
- Sensor startup time < 60 s.
- Data evaluation directly on the device (no cloud computing).
- Visual indicator of the classification result on the device.
- Possibility for one-handed operation.
- Connection to a smartphone where measurement reports can be created and saved.

Additionally, hardware costs should be kept as low as possible. Since no commercially available device could meet all the criteria listed above, a custom sensor solution had to be developed.

#### 3.1.1. Choosing the Most Suitable Non-Destructive Measurement Technology

As a first step, different non-destructive optical measurement techniques were investigated regarding their suitability for the measurement of THC in cannabis samples. This included NIR and MIR measurements in reflection geometry, MIR spectroscopy in attenuated total reflection geometry (ATR-MIR), and Raman spectroscopy with different excitation laser wavelengths (785 nm and 830 nm). To test the suitability of the different measurement techniques, a small sample set of 17 ground cannabis samples with THC concentrations ranging from 0.07 wt% to 14.0 wt% were measured (five measurement spots per sample) using each measurement technique in a laboratory environment. Subsequently, a PLS regression model was calculated for each data set to give an estimation of predictive performance for each technique based on the root mean squared error of cross-validation (RMSECV) for a leave-one-out cross-validation.

The best results were achieved using MIR-ATR spectroscopy with a RMSECV of approximately 0.46 wt%, while MIR measurements in reflection geometry did not reveal a good correlation, most likely due to the low intensity of the back-reflected light. All MIR measurements were carried out on a Lumos FT-IR Microscope (Bruker, Ettlingen, Germany).

In the case of Raman spectroscopy, it was found that with the used table-top Raman devices (Wasatch photonics, Morrisville, NC, USA), the chemical information from the samples was mostly overshadowed by their fluorescence for both excitation laser wavelengths, and no good correlation with the samples’ THC content could be found in the acquired data.

Furthermore, a broad-band, compact FT-NIR spectrometer module based on micro-opto-electronic mechanical systems (MOEMS) technology (Hamamatsu Photonics, Hamamatsu, Japan) [30], already successfully utilized for NIR-based inline-process monitoring in the past [31], in combination with a fiber-coupled reflectance probe (Ocean Optics, Orlando, Florida, USA) and halogen light source (Avantes, Apeldoorn, The Netherlands) was tested for the same sample set and revealed a RMSECV of 0.53 wt%, comparable to what could be achieved with the MIR-ATR setup.

Since MIR-ATR measurements through transparent plastic bags, which was defined as a requirement by the Austrian authorities, is not feasible due to the low sample penetration depth, NIR spectroscopy was ultimately chosen as the most suitable measurement method for the classification of cannabis samples. After testing multiple devices from different manufacturers in the laboratory, cost-efficient MOEMS-spectrometer modules based on tunable Fabry-Pérot filters (NIRONE-X, Spectral Engines, Steinbach, Germany) were identified as best suited, due to their ultra-compact form factor of only 16 × 32 × 35 mm³ and low hardware price.

It was also found during the laboratory phase that, especially for unhomogenized cannabis samples such as untreated whole flower buds, the discrepancy between consecutive measurements on different spots of the sample was significant. To partly counteract these inhomogeneities found in the investigated samples, it was decided to utilize two modules for the measurement, which measure different spots on each sample. This significantly increases the chances for correct sample classification, as also discussed below in Section 3.2.1.

#### 3.1.2. Electrical Design

**Figure 1:**
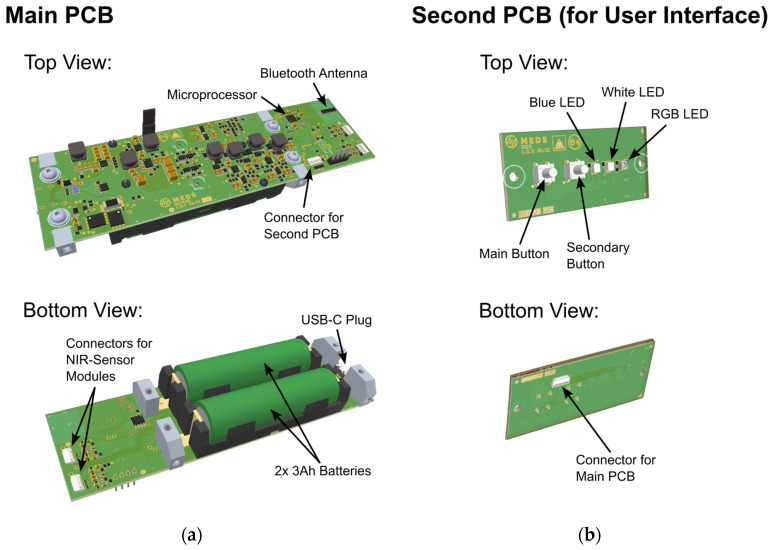
(**a**) Top and bottom view of the main PCB used in the developed sensor. The most important parts are indicated. (**b**) Top and bottom view of the second PCB used for interaction with the user, with indication of the most important parts.

Dedicated electronics were developed to provide electrical power and control for the implemented sensor modules, as well as to carry out necessary calculations directly on board the sensor. The sensor electronics were split into two separate printed circuit boards (PCBs) connected via a ribbon cable, where the smaller one is used for user input. Both PCBs are shown in Figure 1a,b, respectively, wherein the most important parts are also indicated.

The main PCB hosts most of the electronics, including the microprocessor (STMicroelectronics, Plan-les-Ouates, Switzerland) used for data handling and processing, the 2.4 GHz Bluetooth antenna (Johanson Technology, Camarillo, CA, USA) for communication with the smartphone and dedicated app, and two 3 Ah batteries (Samsung, Suwon, South Korea) including power management electronics, which can be charged via the USB-C plug. The two NIR sensor modules are each connected to the main PCB via an 8-pin ribbon cable.

The second PCB hosts two buttons for user interaction as well as three LEDs used for status indication and indication of the measurement result with no smartphone present. A white LED is used to indicate charging and battery status, a blue LED for Bluetooth connection status and an RGB-LED to indicate sensor and measurement status and, most importantly, the measurement outcome after the measurement is finished. Three colors are used to indicate the measurement outcome to the user:

1. Green color indicates that the measured sample contains less than the threshold value of THC.
2. Red color indicates that the sample contains more than the threshold value of THC.
3. Yellow color means that the measurement outcome is uncertain.

If a smartphone is connected to the sensor, the result will also be shown on the smartphone screen in addition to the LED directly on the sensor.

**Figure 2:**
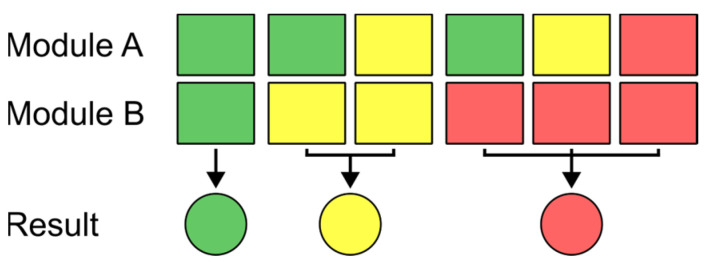
Logic of how the final measurement result, which determines the color shown on the sensor’s RGB-LED (represented by the colored circles) is determined for different results of the two NIR sensor modules.

Since there are two NIR sensor modules in each of the devices which measure different spots on the investigated sample, the result of the measurement of the two sensors might be different due to inhomogeneities in the sample composition, especially for unhomogenized samples. To get a single output, which is later communicated to the user via the RGB-LED, a decision logic has been defined, which determines the overall outcome of the measurement for each combination of results from the two sensor modules and thus the color of the RGB-LED. This logic is schematically shown in Figure 2, wherein the colors green, yellow, and red define the outcomes of the individual sensors as unsuspicious (below the defined THC threshold value), uncertain, and suspicious (above the defined THC threshold value), respectively. The outputs of the individual NIR sensor modules are calculated from the acquired spectra via PLS-DA, which is described in more detail in Section 3.2.1.

#### 3.1.3. Mechanical Sensor Design

**Figure 3:**
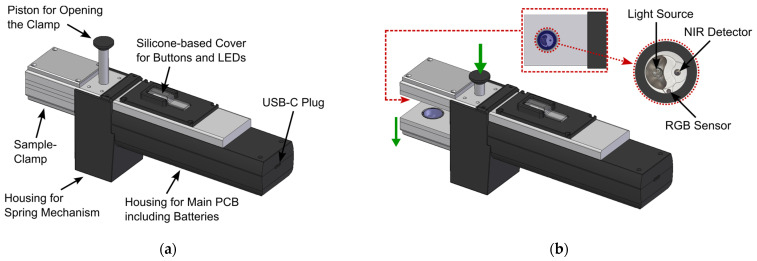
(**a**) CAD-drawing of the final sensor design with closed sample clamp. The different parts of the sensor are labelled in the drawing; (**b**) CAD drawing of the sensor with opened sample clamp. The inset shows a more detailed view of the optical sensing interface on the top part of the sample clamp.

To allow non-expert users to reliably conduct measurements with the developed NIR-based sensor in the field, it was important to establish a mechanical design that can incorporate the necessary NIR sensor modules, user interface, and electronics in a compact and rugged design. Moreover, the sensor should be usable with only one hand, to allow users to carry out additional tasks with their other hand. For this reason, a spring-powered mechanical clamping mechanism was designed that ensures proper sample presentation to the NIR spectrometer modules during the measurement, i.e., good contact between sample and optical sensor interface and restriction of sample movement during the measurement, since such movement can lead to artefacts in the acquired NIR spectra. The final design of the sensor with closed sample clamp is depicted in Figure 3a, where the most important parts are also labelled. Figure 3b shows the sensor with opened sample clamp and offers a more detailed view of the optical sample interface.

**Figure 4:**
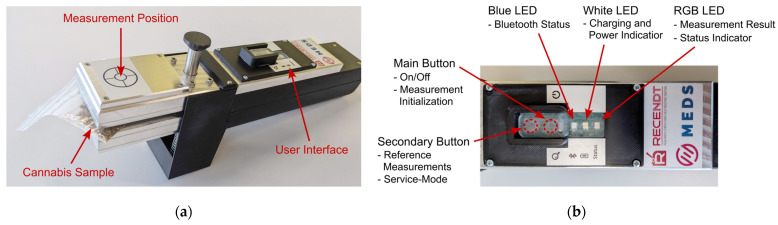
(**a**) Photograph of the sensor prototype with an inserted cannabis sample. The crosshairs sticker on the top indicates the position of the measurement on the inserted sample; (**b**) Detailed view of the user interface on top of the sensor. Buttons and LEDs and their main functions are indicated.

The clamping mechanism can be opened by pushing down the opening piston with the thumb, as also indicated in Figure 3b, revealing the two identical optical sample interfaces on the top and bottom sides of the sample clamp. With the clamp opened, the cannabis sample, stored inside a transparent plastic bag, can be inserted into the clamping mechanism. Once the thumb-piston is released, the built-in springs will close the clamp, firmly holding the investigated sample in place. To help the user with sample positioning, the measurement position is indicated via a crosshairs-sticker on top of the sample clamp, which can be seen in the photograph of the sensor with an inserted cannabis sample shown in Figure 4a. In Figure 4b, a photograph of the user interface on the top of the sensor is depicted, where the transparent silicone-based button and LED cover can also be seen, which protects the underlying PCB from water and dust.

The developed sensor design provides a safe housing for all the sensor components, while also providing single-hand usability once the sample of interest has been inserted into the clamping mechanism.

#### 3.1.4. Conducting a Measurement

**Figure 5:**
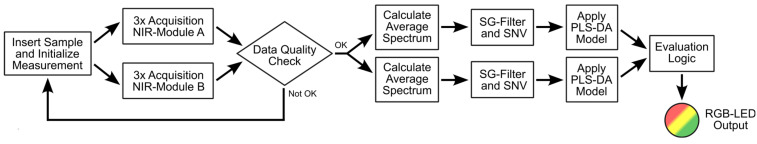
Illustration of the most important steps in the measurement process.

The most important steps in the workflow necessary for the classification of a sample are shown in the diagram in Figure 5. After a sample is inserted into the sensor, the measurement is initialized by pushing the measurement button on the top of the sensor and the RGB-LED starts slowly blinking yellow to indicate an ongoing measurement. First, both sensor modules simultaneously acquire three spectra each on two opposite sides of the sample. These three spectra are checked for consistency by calculating the mean of their wavelength-wise standard deviation. The resulting value must be lower than a predefined threshold, otherwise it means the sample has been moved inside the sensor resulting in unwanted measurement artefacts. Additionally, the mean intensity of the acquired spectra is compared to a predefined lower limit to ensure proper sample presentation and SNR. If either of those criteria is not met, the sensor indicates to the user that the sample needs to be re-inserted into the sensor (fast blinking yellow RGB-LED). If the spectral data pass this data quality check, the three measured spectra are averaged for each sensor module and absorbance spectra are calculated using the internally stored white reference (one for each sensor module). The resulting absorbance spectra are then preprocessed using a Savitzky-Golay filter with subsequent SNV normalization (see also Section 2.3.1) before the PLS-DA model is applied to each spectrum. The PLS-DA model provides a classification output for each sensor module (see Section 3.2.2 for details), which is evaluated using the logic described in Figure 2. The final measurement output is then communicated to the user via the RGB-LED’s color (green, red, or yellow, as described above in Section 3.1.2).

### 3.2. Measurement Results

#### 3.2.1. PLS-DA Model Calibration

**Figure 6:**
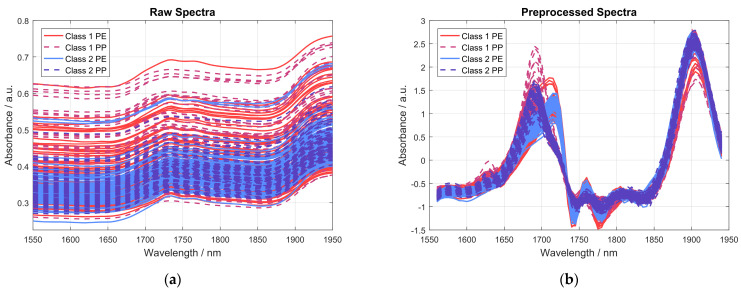
Graphs showing the spectra of 20 exemplary samples, each measured with four different NIR sensor modules, each measured in a PE and a PP bag, resulting in a total of 160 spectra. (**a**) Raw absorbance spectra; (**b**) spectra after preprocessing using a first derivative via Savitzky-Golay filter and SNV normalization.

To successfully distinguish the acquired NIR spectra with respect to THC concentration, the acquired spectral data had to be preprocessed to reduce unwanted interference, as described in Section 2.3.1. Figure 6 shows the spectra of 20 different samples of each class, measured using four different NIR sensor modules, each in a PE and a PP bag, resulting in a total of 160 spectra. The spectra are grouped in four different categories, according to whether the sample has more than, less than, or equal to 0.4 wt% THC, and whether it was measured in a PP or PE bag. Figure 6a shows the raw absorbance spectra while Figure 6b shows the same spectra after preprocessing by a first derivative using a Savitzky-Golay filter and SNV normalization.

**Figure 7:**
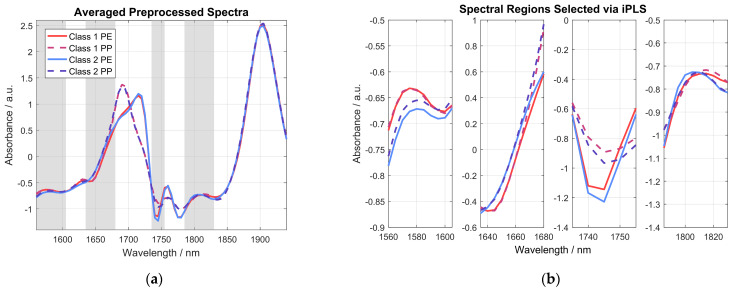
(**a**) Graph showing the averaged data for the sample spectra shown in Figure 6b. The spectral regions with a grey-colored background were determined using iPLS and indicate the most important spectral regions for distinguishing between samples of the two different classes. (**b**) Graph providing a closer look at the four grey wavelength regions chosen by the iPLS algorithm.

A comparison of raw absorbance spectra and preprocessed spectra shown in Figure 6 nicely highlights the importance of the spectral preprocessing, as most of the unwanted interference in the acquired spectra can effectively be removed. However, it is obvious that most of the differences in the acquired spectra, even after spectral preprocessing, are caused by the type of plastic bag the sample was measured in, i.e., PE or PP. This can be seen even more clearly in Figure 7, where the averaged preprocessed spectra shown in Figure 6 for each of the indicated spectra categories are presented. Therein, the most important spectral regions for distinguishing between samples belonging to class 1 (≤0.4 wt% THC) and class 2 (>0.4 wt% THC) are also indicated by a grey-colored background.

These spectral regions were identified using iPLS with an interval size of five variables on the whole calibration data set using a 90/10 cross-validation. For a dedicated band assignment of pure cannabis, the reader is referred to [14]. As clearly visible in the averaged preprocessed spectra, the region between 1680 nm and 1735 nm is dominated by the influences of the chosen plastic bag and is therefore excluded by the iPLS and thus not considered in the calibrated PLS-DA model. Also, the spectral region above 1830 nm is excluded from the model, as it is mostly influenced by the water absorption band, thus sample moisture, and not the sample’s THC content. A closer look at the grey-colored areas chosen by the iPLS algorithm is provided in the four graphs in Figure 7b. The spectral region from 1560 nm to 1605 nm (leftmost graph) shows an almost perfect match between class 1 samples measured in PE (solid red line) and PP (dashed red line). This confirms the absence of influences from the plastic packaging in this spectral region. However, a slight offset is visible in the class 2 samples in the same region for different packaging. This can be attributed to the fact that different positions were measured in the respective samples, which is due to spatial inhomogeneities. This effect is much stronger in the samples of class 2, due to the high spread of THC concentrations (between 0.45 wt% and 14.8 wt%), compared to class 1 samples (between 0.09 wt% and 0.38 wt%), leading to the observed differences in the acquired mean spectra for class 2. The mean preprocessed spectra of the two classes shown in the second graph in Figure 7b match nicely for both types of packaging until approximately 1660 nm. At longer wavelengths, the spectra for each class start to drift apart until they are dominated by the type of packaging material above 1680 nm and are hence no longer considered by the iPLS algorithm. There is a significant difference between PE and PP packaging in the spectral region shown in the third graph in Figure 7b. However, class 2 samples on average have a lower signal in this region than class 1 samples, for both packaging materials. Spectra shown in the fourth wavelength region chosen by the iPLS algorithm, shown in the rightmost graph in Figure 7b, seem to be mainly influenced by sample THC content, especially around 1820 nm, while the packaging still shows some influence on the signal at shorter wavelengths. The wavelengths chosen by the iPLS algorithm were used for the PLS-DA modelling without any additional editing to avoid user bias when it comes to wavelength selection.

**Figure 8:**
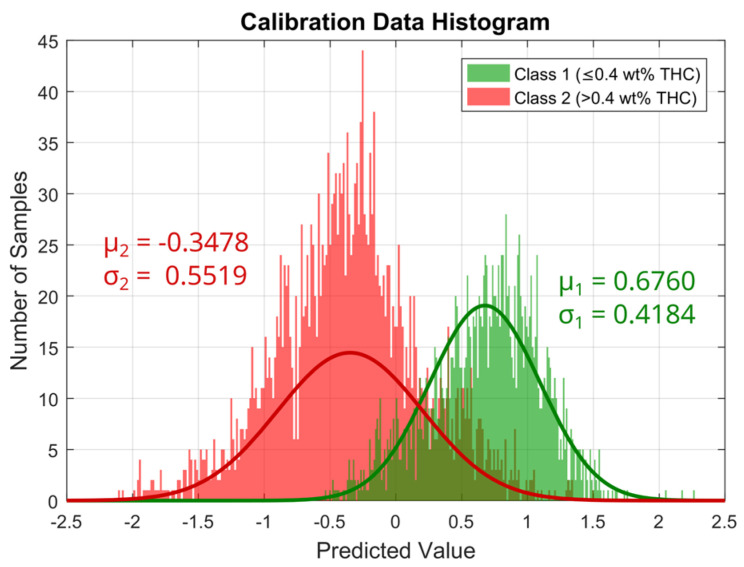
Histogram of the predicted values of the PLS-DA Model for class 1. Predicted values for class 1 samples are shown in green and for class 2 samples in red. The solid line in the respective color gives the normal distribution fitted to the data.

A PLS-DA model was calibrated using 4221 spectra from the calibration data set, which are composed of 42.1% class 1 and 57.9% class 2 spectra. A total of 39 spectra were considered outliers based on their high Q residuals and Hotelling T² values. The number of latent variables (LVs) for the model was determined using 90/10 cross-validation, which revealed six LVs to be the optimal number with the minimal number of misclassifications. The calibrated PLS-DA gives two regression vectors, one for each class, which in the case of a binary model have equal entries but with an opposite sign. A histogram of the predicted values of the calibrated PLS-DA model for class 1, i.e., the scalar product of the regression vector of class 1 and the preprocessed calibration spectra [32], is shown in Figure 8, wherein a clear separation between the spectra of the two classes can be seen. A normal distribution with the means and standard deviations calculated from the predicted values of the calibration spectra is assumed to describe the distribution of the expected predicted values of both classes under consideration. The resulting Gaussian curves (normalized with respect to the integrated area) are also indicated in Figure 8 along with the calculated means and standard deviations for each class.

These Gaussian curves are used to calculate a classification probability for each individual predicted value based on Bayes theorem, which leads to a classification probability for each of the two classes for each sample [33]. Since we use a binary classification, the classification probability for the two classes for each sample sums up to 1.

However, there is a non-negligible overlap between the two classes, caused by a high degree of similarity between the samples of class 1 and class 2, especially close to the set threshold of 0.4 wt% THC. In total, approximately 14.9% of the calibration spectra are misclassified by the PLS-DA model when each spectrum is assigned the class with higher probability. To further reduce the number of false classifications, only those classifications with a probability of more than 60% are considered as legal or illegal, and all other classifications are considered uncertain, which affects a total of 6.4% of the spectra in the calibration data set. Of the remaining 93.6% of spectra that are classified with a probability of 60% or more, only approximately 13.0% are misclassified.

#### 3.2.2. PLS-DA Model Validation

To validate the calibrated PLS-DA model, the two developed sensor prototypes were handed to employees of the Austrian authorities, who, after short introduction on sensor operation, measured a total of 83 samples, each with both sensors and once in a transparent PE and once in a transparent PP bag. The validation sample set contains 27% class 1 samples with ≤0.4 wt% THC and 73% class 2 samples with >0.4 wt% THC as determined using GC reference measurements. Spectral data were acquired over the course of two months by different users. The prediction results of the PLS-DA model applied to the calibration data set is summarized in Table 1.

Of all the measured sample spectra, approximately 10.6% are classified with a probability of less than 60% and are thus considered uncertain. This amounts to a total of 70 spectra, which are made up of 27% class 1 samples and 73% class 2 samples, which is the same split as for the overall validation data set, hinting at a non-biased prediction of the calibrated model. Moreover, 66% of these uncertain predictions pertain to samples within ±0.2 wt% of the THC threshold of 0.4 wt%. Of the remaining samples, which are classified with a probability of 60% or higher by the PLS-DA model, approximately 79% are classified correctly, whether all samples or only the individual classes are considered.

**Figure 9:**
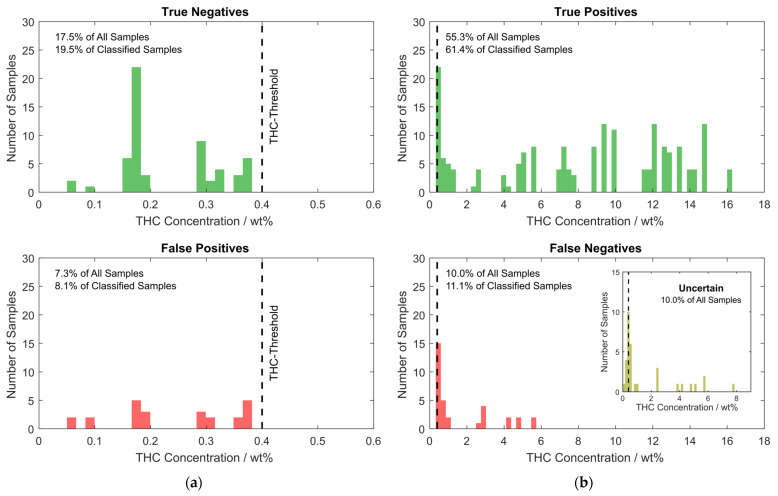
Histograms showing the distribution of samples in the validation data set with respect to THC content. The bar height represents the number of samples contained within the concentration range given by the bar width. (**a**) Class 1 samples. On top, the correctly classified class 1 samples are shown as true negatives (correctly classified as non-suspicious by the device). On the bottom, the false positives (class 1 samples misclassified as class 2 by the device) are shown; (**b**) Class 2 samples. On top, true positives (class 2 samples correctly classified as suspicious) and on the bottom, false positives (class 2 samples incorrectly classified as non-suspicious) are shown. The inset shows the histogram of the samples with uncertain outcome.

However, the numbers shown above consider each of the NIR sensor modules individually, but two sensor modules are built into each handheld sensor, which measure two different spots on the sample simultaneously. The two spectra acquired with these sensors belong to the same sample and are classified according to the logic shown in Figure 2. If the two simultaneously acquired spectra are not treated individually, but according to this logic, which also determines the color of the RGB LED on the device that indicates the result to the user, the outcome is slightly different. These results are summarized in the histogram in Figure 9.

By considering both spectra measured on each sample using the logic from Figure 2, the percentage of uncertain predictions is almost unchanged at 10.0%, while correct classifications could be increased to approximately 72.8% and wrong classifications could be decreased to 17.2%. Of the 90% of samples that are reliably classified with a classification probability of at least 60% according to the PLS-DA analysis, approximately 81% are correctly classified. The inset histogram at the bottom in Figure 9b nicely shows that, for the case where individual sensor modules are considered, most of the uncertain predictions result from samples that have a THC concentration close to the threshold of 0.4 wt% THC. Since the sample is classified as class 2 if the spectrum of any of the two sensors is classified as class 2 with a probability of at least 60%, a higher percentage, namely 84.7%, of class 2 samples are correctly classified. On the flip side, the fraction of class 1 samples that are correctly classified is reduced to 70.7%. As evident from the histogram in Figure 9b, most class 2 samples (58%) mistaken for class 1 have a low THC content of below 0.6 wt% and no sample with a THC content of more than 5.7 wt% is misclassified. The misclassified samples are almost equally distributed between the two tested devices (53% vs. 47%) and samples measured in PP or PE bags (46% vs. 54%), rendering a systematic error unlikely.

## 4. Discussion and Conclusions

The developed NIR-based device allows the user to distinguish between suspicious and non-suspicious cannabis samples in the field in a quick, reliable, and non-destructive manner. The device can be operated with one hand and allows data evaluation directly on the device’s hardware, without the necessity of internet connection for cloud computing, significantly increasing its field usability as well as data security. The measurement result is directly displayed to the user via an RGB-LED and optionally on a connected smartphone running a custom app to display additional information and create measurement reports for later reference. An important benefit regarding field application is the ability to measure cannabis samples directly within transparent plastic bags made from polypropylene or polyethylene, which make up the majority of the cannabis packaging encountered in the field.

By using two individual NIR sensor modules that measure simultaneously at two different spots on the cannabis sample with subsequent evaluation according to the logic shown in Figure 2, the number of correct classifications could be increased in comparison to measurements considering only a single measurement spot. Moreover, the application of two NIR sensor modules ensures that, when being used by Austrian authorities in the field, only a small percentage of illegal cannabis samples with more than 0.4 wt% THC are misclassified as non-suspicious samples, due to the higher chance of catching a THC-rich area enabled by data acquired from two different spots on the sample. Notably, a few samples with rather high THC content of up to 5.7 wt% are also misclassified. This is mostly attributable to inhomogeneities in the samples, which can only be partly counteracted by using two sensor modules to measure two spots simultaneously. If only the data from a single sensor module are evaluated, some samples with up to 7.8 wt% are misclassified as unsuspicious, strengthening the rationale for the use of two sensors. To achieve a lower percentage of falsely misclassified samples, either multiple measurements of a single sample or well-defined sample preparation procedures would be necessary. However, for better field-applicability, the measurement procedure was kept as simple as possible. Even if that means a slightly lower classification accuracy, the sensor still provides a substantial improvement compared to pure subjective assessment of the nature of encountered cannabis samples.

At the same time, a significant number of legal samples are correctly classified as non-suspicious, which can drastically reduce the number of confiscated legal samples, leading to considerable monetary savings for the authorities.

The used hardware components are suitable for implementation of common machine learning models for sample classification, e.g., random forest or support vector machine algorithms, which can potentially increase the number of correct classifications; this is part of ongoing research. Apart from application for cannabis classification, the method is generic, i.e., it can be adapted to other drug species by adjusting spectral ranges and developing specific chemometric models.

## 5. Patents

The developed measurement solution for untreated cannabis samples through transparent plastic packaging was filed at the Austrian patent office under patent number A50110/2023 on 20.02.2024.

## Figures and Tables

**Table 1:** Prediction results of the PLS-DA Model applied to all measured spectra of the validation data set.

Samples	Correct Classifications	Wrong Classifications	Uncertain Predictions
All measured samples	70.4%	19.0%	10.6%
Excluding uncertain predictions *	78.7%	21.3%	-
Class 1 samples (≤0.4 wt% THC)	79.1%	20.9%	-
Class 2 samples (>0.4 wt% THC)	78.6%	21.4%	-

* Samples that are classified with a probability of ≥60%, which is the case for 89.4% of the samples.

## Data Availability

The data that support the findings of this study are available from the corresponding author, R.Z., upon reasonable request.
